# Towards greater transparency in neurodevelopmental disorders research: use of a proposed workflow and propensity scores to facilitate selection of matched groups

**DOI:** 10.1186/s11689-020-09321-6

**Published:** 2020-07-24

**Authors:** Janet Y. Bang, Megha Sharda, Aparna S. Nadig

**Affiliations:** 1 Department of Psychology, Stanford University, 450 Serra Mall, Stanford, CA 94305 USA; 2 International Laboratory for Brain, Music and Sound Research (BRAMS), Université de Montréal, 90 Avenue Vincent D’Indy, Montreal, QC H2V 2S9 Canada; 3 School of Communication Sciences and Disorders, McGill University, 2001 Avenue McGill College Suite 800, Montreal, QC H3A 1G1 Canada; 4 Centre for Research on Brain, Language & Music, 3460 de la Montagne, Montreal, QC H3G 2A8 Canada

**Keywords:** Matching, Transparency, Group comparison, Reproducibility, Propensity score, Covariate

## Abstract

**Background:**

Matching is one commonly utilized method in quasi-experimental designs involving individuals with neurodevelopmental disorders (NDD). This method ensures two or more groups (e.g., individuals with an NDD versus neurotypical individuals) are balanced on pre-existing covariates (e.g., IQ), enabling researchers to interpret performance on outcome measures as being attributed to group membership. While much attention has been paid to the statistical criteria of how to assess whether groups are well-matched, relatively little attention has been given to a crucial prior step: the selection of the individuals that are included in matched groups. The selection of individuals is often an undocumented process, which can invite unintentional, arbitrary, and biased decision-making. Limited documentation can result in findings that have limited reproducibility and replicability and thereby have poor potential for generalization to the broader population. Especially given the heterogeneity of individuals with NDDs, interpretation of research findings depends on minimizing bias at all stages of data collection and analysis.

**Results:**

In the spirit of open science, this tutorial demonstrates how a workflow can be used to provide a transparent, reproducible, and replicable process to select individuals for matched groups. Our workflow includes the following key steps: Assess data, Select covariates, Conduct matching, and Diagnose matching. Our sample dataset is from children with autism spectrum disorder (ASD; *n* = 25) and typically developing children (*n* = 43) but can be adapted to comparisons of any two groups in quasi-experimental designs. We work through this method to conduct and document matching using propensity scores implemented with the R package MatchIt. Data and code are publicly available, and a template for this workflow is provided in the Additional file 1 as well as on a public repository.

**Conclusions:**

It is important to provide clear documentation regarding the selection process to establish matched groups. This documentation ensures better transparency in participant selection and data analysis in NDD research. We hope the adoption of such a workflow will ultimately advance our ability to replicate findings and help improve the lives of individuals with NDDs.

## Introduction

Neurodevelopmental disorders (NDDs) manifest early in life and can present challenges to one’s daily functioning in domains such as language and cognition, which can in turn interfere with access to social, academic, and economic opportunities [1]. These disorders include autism spectrum disorder (ASD), intellectual disability (ID), attention-deficit/hyperactivity disorder (ADHD), motor disorders, and speech sound disorders, to name a few. NDDs are complex, and within many NDDs, there is wide genetic and phenotypic variation. A common goal of research is to explain the processes underlying this variation; such findings can inform clinical and educational practice and thereby optimally support individuals with NDDs.

In studies of individuals with NDDs, researchers often compare two or more groups by employing *matching*. When researchers match two or more groups, they will try to “control” for or balance on pre-existing variables that differ between groups (e.g., age or language level). This matching is important because when two or more groups differ on pre-existing characteristics other than diagnosis, these pre-existing differences limit our ability to link group diagnosis to performance on an outcome variable. In practice, this matching can be difficult to achieve. There are multiple considerations such as the number of available participants, whether variables can or should be matched if they are inherent to one group [2], and proposed cutoffs of inferential statistics for a “well-matched” group [3, 4]. However, one consideration of matching has received relatively little attention in the NDD literature, and it is critical because it occurs early in the process of matching groups: the selection of participants to establish well-matched groups.

The process of selecting participants often goes undocumented [4], but it is an early step prone to unsuspecting bias. If matching depends on certain considerations that are left unknown for future researchers, this bias then limits replicability of findings. We illustrate a workflow to improve transparency when matching and bring attention to the use of propensity scores to facilitate the selection of participants for matched groups. Group matching is one area that can particularly benefit from the growing awareness of transparency, particularly given the concerns of reproducibility (producing the same result given the original data and access to methods) and replicability (being able to replicate the results of a study with a new dataset) in psychological science [5, 6]. The selection of participants is especially critical given that it is one of the first decisions after data collection before researchers can clearly analyze and interpret study outcomes. In this article, we present a guided step-by-step tutorial with open access to the original data and supporting R code [7, 8] to demonstrate group matching using propensity scores.

### Matching as a method in NDD research

In research on NDDs, individuals cannot be randomly assigned to a group with or without an NDD. Instead, groups are pre-determined based on clinical symptoms, resulting in a *quasi-experimental* design. In contrast to a quasi-experimental design, an *experimental* design with true randomized assignment better enables one to draw a causal link between the group assignment and performance on the outcome variable, regardless of pre-existing differences between groups. For example, say you randomly assign high school students to either one intervention that provided apples as a snack (group A) versus another intervention that provided bananas (group B); the outcome variable measured the number of times students participated during their classes. You found that group A participated more; therefore, you conclude that apples increased class participation. However, you realize after you randomly assign the groups that group A was slightly older than group B. Yet because of randomization, how age might have influenced participation could be attributed to random error, rather than being highlighted as an alternative or competing reason for why group A participated more often^1^. However, in a quasi-experimental design, there is no randomization. Instead, you are sampling from two categorized groups. For example, in a quasi-experimental design, you might compare participation between students in traditional schools versus students in year-round schools; in this design, both groups were established by sampling from two different populations. Both populations may differ on multiple pre-existing characteristics that could be related to class participation such as age, attention, or motivation, in addition to their pre-determined grouping of school status. The consequence of a quasi-experimental design is that when you compare performance between groups, it is difficult to disentangle whether school status or other pre-existing differences between groups can explain the outcome (e.g., class participation). The goal with matching is to minimize the extent of differences between groups such that you can make clearer inferences about how group membership alone relates to performance on outcome variables.

In individuals with NDDs, there is often wide variability along numerous characteristics that are not a central part of the diagnostic criteria. Therefore, we want to minimize differences between groups when those characteristics may be related to both group diagnosis and outcome variables. When such pre-existing characteristics link with outcome variables, they are referred to as *covariates*. While there is a danger of covariates in both true experimental and quasi-experimental designs, the nature of quasi-experimental designs means that covariates must be addressed with a method other than randomization [9]. One method to mitigate the role of covariates is matching. Notably, if differences are defining and inherent to one group versus another, there may not be a need to address those differences. For example, children with ADHD are defined in part by attention difficulties; therefore, we would expect group differences on pre-existing variables of attention. More about matching in NDDs and other quasi-experimental designs can be seen in Blackford (2007) [9] and Stuart and Rubin 2008 [10]. For ease, we refer to a design with two groups for the remainder of this article.

### Selection of matched groups

The realities of sampling often result in groups that differ on variables other than the diagnosis of the NDD in question, leaving researchers with the task of using a subset of samples from each population to achieve matched groups. The term *selection bias* refers to the bias introduced when groups are not randomly selected, thus resulting in one group having different characteristics than the other [10]. Selecting participants to form matched groups occurs either during or after the stage of data collection. When selecting participants, researchers ideally decide a priori on one or more variables to match between groups, decide on a matching criterion, choose which children to include, and then conduct statistical tests to confirm if groups are matched based on balancing criteria such as *p* values, effect sizes, and variance ratios [3, 4, 11]. However, if groups are not matched as closely as one would like according to the balancing criteria, then some individuals are removed and/or new ones are selected to be in the matched group. If there are multiple variables to keep track of, it is difficult to know which individuals to select that will satisfy an overall balance across multiple variables between groups.

There are many difficulties with this selection process. For one, selection of participants is iterative and often undocumented [4], which can make it difficult to replicate decisions for future research. Any iteration may involve numerous decisions, and whether these decisions are arbitrary, biased, or random is unclear. For example, if one wants to match groups on two or more variables that might affect outcomes such as age and IQ, and after data collection the data reveal that groups differ on both age and IQ, then which variable does one begin with to match on? Or should one somehow consider both variables at the same time? This may then lead to another challenge: arbitrary selections. One potential situation in the case of pairwise matching (see next section) is if there are two individuals with the same value on some variable (e.g., same age and/or IQ) in group A that one wants to match with in group B, which individual from group A does one include? A researcher faced with this dilemma may be led to another potential challenge: unsuspecting bias. For example, if two individuals have the same value on some variable (e.g., the same IQ score), and the researcher was involved in testing and knew that one individual appeared more attentive than the other, then which individual gets selected? Choosing the less attentive individual could be perceived as being less biased, but then could unfairly remove an individual who did not have any difficulty with the task. Choosing the more attentive individual would be biasing selection based on some aspect of the individual’s performance. When the possible number of matched group sets can be represented by a combination of nCr,^2^ it is apparent how quickly the number of different matched sets can grow. With a sample of 50 typically developing children to match to a set of 25 children with ASD, the possible subsamples of 25 typically developing children are over 126,000,000,000,000! Some of these sub-samples may be unlikely depending on matching criteria, and not all decisions may be arbitrary or biased. However, the variability among these scenarios illustrates the number of potential undocumented decisions that can limit the replicability of those research findings to other studies with similar samples, delaying the benefits of this research for individuals with NDDs.

### Pairwise versus group matching

There are two possible ways to match groups: pairwise or group matching. In pairwise matching, balanced groups are achieved by ensuring that a particular individual in one group is selected to be within a narrow criterion range that is similar to a specific individual in the other group, for example, ensuring that individuals in each group are selected to be within 3 months in age of each other. This rigorous form of matching is often easier to achieve in larger sample sizes (e.g., *n*s > 50) when there are more possible matches [12, 13], thus retaining more individuals and increasing power without risking reducing a small sample size even further. However, pairwise matching can also be done in smaller samples when sampling is considered during recruitment [14]. Another matching strategy is group matching, where researchers select individuals such that on the desired matching variable, the distribution over the group is similar between groups. One way this can be achieved is by only selecting individuals in a clinical group who are within the same range on the desired variable in the control group (e.g., only including children with an NDD who do not have intellectual disability [15]). As seen in Table 1, the verification of well-matched groups can be achieved using visual analysis of graphical distributions and/or reporting appropriate descriptive or inferential statistics of effect sizes, variance ratios, and *p* values [3, 4]. We discuss next the use of *propensity scores* to remove bias when implementing either pairwise or group matching.

**Table 1:** Visual analysis and statistics to assess group matching

	Graph or statistic
Visual analysis	• Boxplots • Histograms • Density plots • Dot plots
Descriptive statistics	• Means • Standard deviation • Range • Cohen’s *d* (includes both groups) [4, 16, 17] • Variance ratio (includes both groups) [4]
Inferential statistics	• *t* tests or Kolmogorov-Smirnov test [18] (continuous variables) • Chi-square test [19], Fisher’s exact test [20], or Wilcoxon test [21] (categorical variables)

### Propensity scores to reduce selection bias

The use of propensity scores has been demonstrated in multiple fields including social work [22, 23], medicine and public health [24–26], and economics [27]. While support for the use of propensity scores in NDDs has been around for more than 10 years [9, 28], their application is still uncommon in research on NDDs. Propensity scores were introduced by Rosenbaum and Rubin [29] and are created by summarizing multiple variables into a single scalar score for each participant. There are multiple parametric models that are used to calculate propensity scores. Logistic regression is one commonly used option where the dependent variable is the binary grouping variable [28, 30, 31]. Each individual’s propensity score is derived from a model that includes all covariates and predicts group assignment; that is, the score is the probability of assignment to either group [9, 29]. An individual’s propensity score represents an estimated distribution of the included covariates as if the observed covariates came from the same multivariate distribution across groups [31]. One limitation of using propensity scores includes sample size requirements. Another drawback is that they are less frequently used in research on NDDs (but see [9, 28] for some examples), making it difficult to understand the broader scope of benefits and consequences of such methods. Although some authors suggest large sample sizes are required [4], others indicate that 5–10 participants per covariate based on the sample size in the main group of interest may be sufficient [9]. A more in-depth discussion of propensity scores can be seen in Blackford [9].

Once a propensity score is calculated, there are multiple ways to utilize this score, although we focus here on how they can be used to facilitate selecting individuals to match groups [29].^3^ One main benefit is that multiple covariates can be represented in a single score, thus facilitating matching when there are two or more covariates under consideration [10]. Propensity scores can also be used as a covariate in regression analyses, thus utilizing a single score to represent two or more scores in their contribution to the outcome variable [9, 31]. Second, researchers can utilize statistical packages to calculate propensity scores and establish matched groups, ensuring better documentation during the selection process.

### Current study

In sum, matching is a common method used to make inferences about individuals with NDDs. These inferences rely on reproducible and replicable decision-making in one of the earliest steps in data analysis: the selection of matched groups. This article details our proposed workflow to encourage transparency when establishing matched groups and demonstrates how to use propensity scores to facilitate this process. For those new to matching in NDD research, our goal is to raise awareness of the issues that can occur when selecting participants for group matching. For researchers so inclined, we provide a workflow that can be applied to their own data and then be shared, thus supporting broader open science goals of transparency, reproducibility, and replication [5]. Importantly, we emphasize that what we share is a *workflow*. This means that though we implement this in R, researchers who do not use analytic tools with shareable code can still document their decision-making processes and make these available as a text document in public repositories for open science (e.g., https://osf.io/). We use data from a quasi-experimental study of referential gaze processing by children with ASD (*n* = 25) or typical development (*n* = 43) in the context of word learning and action learning to demonstrate this workflow [34, 35].

## Methods

### Sample

Participants were 6- to 11-years old and included 43 typically developing (TD) children and 25 children with ASD. Children had English or French as their dominant language. Based on parent report, TD children did not have developmental, learning, or behavioral disorders, nor did they have physical, vision, or hearing limitations that would interfere with study procedures. TD children also did not have any first- or second-degree relatives with ASD. Five additional TD children were tested but excluded according to pre-determined study criteria because of participation in an earlier version of the experiment (3), a hearing aid (1), and a diagnosis of ADHD (1). Children with ASD did not have any other medical conditions associated with ASD (e.g., fragile X syndrome) and no physical, vision, or hearing limitations that would interfere with study procedures (e.g., color blindness). Eight children with ASD were diagnosed with comorbidities, including ADHD, speech dyspraxia, or language impairment. Three additional children in the ASD group were tested but were excluded because they were unable to complete the study (2) or did not meet criteria for ASD (1). All parents provided informed consent and children provided informed assent prior to study participation.

### Measures

All participants completed a standardized assessment battery on nonverbal IQ, language abilities, and social skills. We assessed nonverbal IQ using the composite score of the Leiter International Performance Scale, Third Edition (Leiter-3 [36]), which has a mean score of 100 (SD = 15). Language was assessed with scaled scores on the Word Classes, Recalling Sentences, and Word Associations subtests of the Clinical Evaluation of Language Fundamentals—4th Edition (CELF-4 [37, 38]), which have mean scaled scores of 10 (SD = 3). English or French versions were used depending on the child’s dominant language. Children’s diagnoses were confirmed using the Social Communication Questionnaire (SCQ)—Lifetime form [39]. The SCQ is a 40-item parent-report questionnaire where caregivers respond to yes or no questions about their child’s social communication skills before age 5. Children’s social skills were assessed by parent report using the Socialization domain of the Vineland Adaptive Behavior Scales-Second Edition (VABS-II [40]), which has a mean score of 100 (SD = 15).

### Matching methods

Many different tools are available to conduct propensity score matching. These include the Stata commands such as *psmatch2* [41] and *teffects psmatch*, and options in SPSS. A comprehensive list of tools to employ propensity score matching can be found elsewhere [42]. We chose to use the *MatchIt* program [30] because it is implemented in R, a commonly used, open-source statistics software with additional graphing tools. It is also important to note that many tutorials on the subject have been available outside of NDD research [26, 28, 43, 44]. Users new to R can find other helpful documentation of propensity score matching, with additional guided steps of how to install and load packages in R [44].

Within MatchIt, there are many different algorithms that can be used to attain matched groups with propensity scores. To use similar terminology as that used in MatchIt documentation, we hereby use the term *control* to refer to the comparison group and *treatment* to refer to the group with the NDD in question.^4^ The different methods within MatchIt include the following: (1) *exact matching*, where control participants are matched on treatment participants with the exact same value on all covariates; (2) *subclassification*, where the sample is separated into subclasses such that there is a similar distribution of covariates between groups within each subclass; this can be useful when there are many covariates and it is too difficult to establish exact matches; (3) *nearest neighbor*, where the control participant closest in distance is selected for each treatment participant (most similar to pairwise matching); (4) *optimal matching*, where the average absolute distance is minimized across all pairs (similar to group matching); (5) *genetic matching*, where an algorithm is used to find a set of weights for each covariate to best match participants; and (6) *full matching*, where all available participants are used and one or more control participants is selected for each treatment participant [43]. Further detail on the different methods can be seen elsewhere [30, 32]. In this article, we compared *nearest neighbor* and *optimal matching* methods because of their relation to pairwise and group matching methods common to NDD research.

### Proposed workflow

**Fig. 1:**
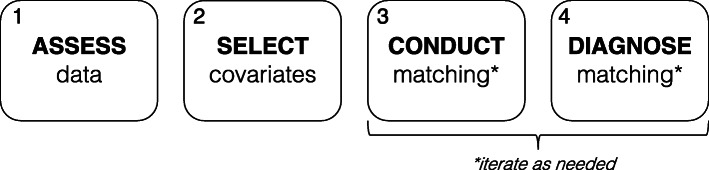
Workflow to achieve matched groups. The four key steps of our proposed workflow to achieve matched groups

Per recommendations [10], this workflow was implemented without any prior knowledge of relations between group membership (ASD vs. TD children) and outcome variables (e.g., looking time to video stimuli, word learning, action learning). As long as one remains unaware of the relation between group and outcome variables, these steps can be iterated until one is satisfied with their matched groups. Adapting prior guidelines [10, 28, 32, 44], we follow the four steps seen in Fig. 1.

#### Step 1: Assess data

This step assesses all potential participants to be included based on the inclusion and exclusion criteria. It also documents any children removed due to pilot testing and data cleaning (e.g., participants must pass practice trials to be included in the study or have a minimum amount of data). Otherwise, all individuals should be included who meet the inclusion criteria and meet the minimum requirement(s) of available data.

#### Step 2: Select covariates

The goal of this step is to be selective of the covariates that will cloud the interpretation of relating group membership and outcomes. One can also visualize the distributions of all potential covariates between groups to carefully examine whether matching on certain covariates may result in lower sample sizes than desired. One should justify the choice of covariates a priori based on prior theory, hypotheses, and/or established relations in the literature [46]. For example, if prior studies have demonstrated that older children with ASD look longer at stimuli, then age may be a covariate in a study examining whether groups differ on an outcome of looking time to visual stimuli. The choice of covariates is not a trivial one [47]. The purpose of matching is to account for confounding variables that are (1) related to group membership and (2) related to the outcome variable. It is important to consider how the covariate is related to the outcome variable and whether it should be considered when matching. For example, in the present study examining referential gaze following, matching on joint attention abilities may lead to a lack of group differences on this measure because joint attention abilities are likely intricately related to referential gaze following; thus, we would not want to match on joint attention abilities.^5^ Moreover, there are arguments against the use of certain covariates widely used in research with individuals with NDDs (e.g., see Dennis et al. [2] for the use of IQ). Because of these challenges, it is critical to be selective and justify these choices prior to analyses in relation to outcomes, particularly because it is not always feasible to match on all potential covariates. There is always the possibility to perform post hoc covariate analyses on additional covariates that were not considered to match groups, although this increases type I error and should be established as exploratory analyses to best guide future studies.

#### Step 3: Conduct matching

This step involves conducting matching according to documented decisions and/or applying matching algorithms.

#### Step 4: Diagnose matching

At this point, you can assess the distribution of group matching on propensity scores and individual covariates using visual analysis such as histograms; dot plots or boxplots; descriptive statistics such as means, standard deviations, effect sizes, and variance ratios; and inferential statistics such as *t* tests and chi-square tests [4, 48]. Steps 3 and 4 can be iterated until matching is satisfactory.

## Results

A publicly available tutorial with the accompanying data, R code, and text can be seen on our GitHub repository [8].

### Step 1: Assess data

The final possible sample size to include is 43 TD children and 25 children with ASD. For reasons outlined in the Sample section, five TD children and three children with ASD were tested but excluded from the study. Data cleaning standards required that children looked at each video for more than 25% of the length of each respective video. After a review of children’s looking time, we removed specific trials for some participants (four possible trials per participant), but this did not result in excluding any additional participants.

### Step 2: Select covariates

Prior to data collection, we considered multiple covariates that could influence children’s performance on the experimental tasks. Ultimately, we could not include all possible covariates given our sample size; thus, we describe how we decided on our final two covariates of age and IQ.

The initial covariates we considered were based on their known relationships with referential gaze following or word learning in experimental tasks. These covariates included age, nonverbal IQ, language ability, sex, and parental education [49–53]. We decided to exclude the covariates of sex and parental education. Though both variables are known to be related to language abilities in the general population [54, 55], we prioritized age, nonverbal IQ, and language ability due to their known relations with referential gaze following and language in children with ASD [52, 56].

**Fig. 2:**
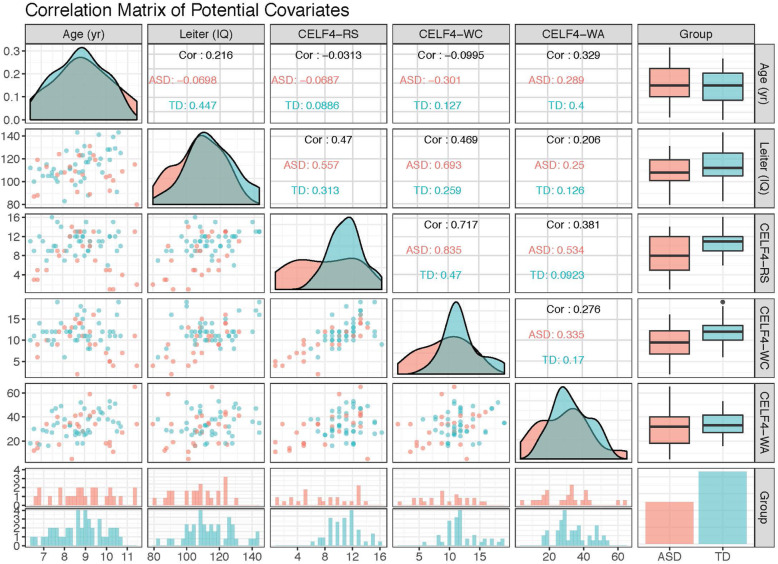
Correlation matrix of potential covariates. ELF-4-RS = CELF-4 Recalling Sentences (scaled scores); CELF-4-WC = CELF-4 Word Classes (scaled scores); and CELF-4-WA = CELF-4 Word Associations (raw scores, because no normative data is provided)

We examined the distributions and interrelations between age, nonverbal IQ, and language abilities seen in Fig. 2, comparing similarities and differences in their distributions between groups. The density plots arranged diagonally and the histograms at the bottom of the figure can be used to compare the distribution of scores in ASD and TD groups. The scatterplots and correlations on either side of the density plots can be used to examine the strength of the relation between variables both within and across groups. The boxplots and bar graphs on the right-hand side provide another way to compare distributions between groups. From these plots, we concluded that while distributions were similar on age and IQ, wide heterogeneity was seen in the ASD group on language variables that were not seen to the same extent in the TD group. Whereas the ASD group ranged widely from 2 SD below the mean to 2 SD above the mean on the normed language measures, there were no children in the TD group 2 SD below the mean. For Recalling Sentences, the distribution of scores in the TD group included 73% of children at or above the mean, whereas this was the case for only 40% of children with ASD. In contrast to the different distributions between groups on Word Classes and Recalling Sentences, the performance of children with ASD and TD children was more similarly distributed on Word Associations. Thus, on measures of semantic language, children with ASD versus TD children demonstrated both similarities (as measured by the Word Associations subtest) and weaknesses (as measured by the Word Classes subtest), whereas a large proportion of children with ASD had weaker structural language abilities (as measured by the Recalling Sentences subtest).

Taken together, these findings indicated that matching on all variables of age, nonverbal IQ, and the three language measures would likely result in less than half of our sample of children with ASD to be matched to a group with TD children. To retain as many children with ASD as possible, we matched on age and nonverbal IQ because groups were similarly distributed on these measures and both covariates have demonstrated relations with our primary experimental manipulation: how children learn with referential gaze. The choice of two covariates followed the guidelines by Blackford, of maintaining an approximate ratio of 5–10 participants per covariate [9].

Though one of our outcomes measures of interest was word learning, and children with stronger language abilities have been shown to have better performance on word learning tasks, we concluded that the overarching goal was to examine how children use referential gaze to learn in two different contexts (word learning and action understanding). Additionally, our experimental manipulation tested how children with ASD treated referential gaze in contrast to an arrow cue, and there was no theoretical or evidence-based rationale suggesting that having stronger language abilities meant that children would learn better with one cue vs. another (or the contrary that weaker language abilities meant that children would learn worse with one cue versus another). Thus, we reasoned that regardless of language ability, it was still possible to test whether children learned new words differently with a referential gaze cue vs. an arrow cue. One benefit of including all children with ASD was that the sample would reflect part of the heterogeneity seen in language abilities (keeping in mind that our sample was selected to have nonverbal IQ in the normal range, and therefore does not represent a full range of language abilities). We decided to further investigate the role of language ability on our experimental measures as a part of our exploratory analyses.

### Step 3: Conduct matching

As seen in Table 2, nonverbal IQ was significantly higher in the TD group (*n* = 43) than in the ASD group (*n* = 25; *p* = .039), although on age, both groups shared similar means and standard deviations (*p* = .570). Our next step was to select participants such that groups were balanced on both age and nonverbal IQ.

**Table 2:** Full sample comparison between TD and ASD groups on age and nonverbal IQ

	ASD (*n* = 25)	TD (*n* = 43)	*p*
Age (years)	8.93 (1.34)	8.75 (1.14)	.570
Nonverbal IQ (Leiter)	107.16 (13.61)	114.53 (14.18)	.039

There are multiple software available across a variety of platforms to match groups [42], and researchers can also document the process of this workflow including this step in writing (see Additional file 1 for examples of what to consider and document). We chose to conduct matching using the MatchIt package [30] in R. We conducted both the nearest neighbor and optimal matching algorithms. Both algorithms resulted in the same 25 TD children chosen as matches to the 25 children with ASD. The nearest neighbor algorithm selects the best match specified by a default distance measure (a logit used when calculating the propensity scores). Matches are chosen one at a time by choosing a control unit that has not yet been matched but is closest to the treatment unit based on the defined distance measure. In contrast, the optimal matching algorithm achieves a matched sample by aiming for the smallest average absolute distance between matched pairs; this method is useful when there may not be appropriate matches for all members of a group. Further details can be seen in Ho et al. [30].

### Step 4: Diagnose matching

**Fig. 3:**
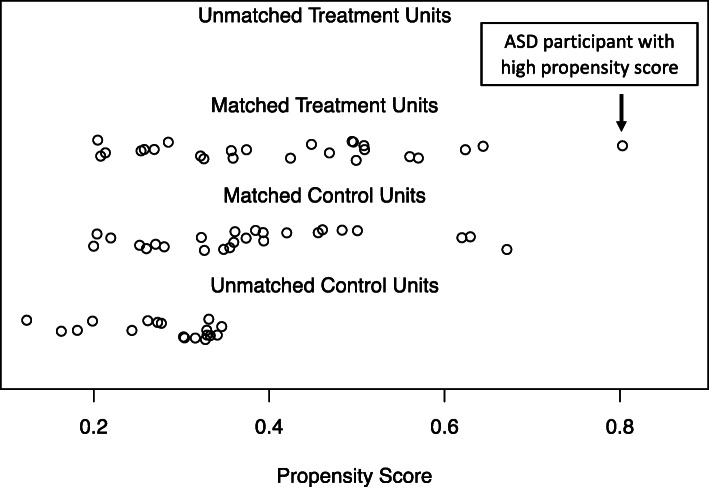
Distribution of propensity scores when including age and nonverbal IQ with the full sample (ASD *n* = 25, TD *n* = 43). Matched treatment units = children with ASD; matched control units = selected matches of TD children; unmatched control units = remaining unmatched TD children. Propensity scores calculated using the nearest neighbor and optimal matching methods resulted in the same values. This plot indicates the distribution of propensity scores when including covariates of age and IQ for all 25 children with ASD and 43 TD children. We see a similar distribution of propensity scores for matched treatment units and matched control units, ranging from scores of 0.2 to above 0.6. Among the matched treatment units, there appears to be one outlier where a child with ASD was assigned a propensity score of approximately 0.8

We first examined how well groups were matched on their propensity scores. As seen in Fig. 3, visual inspection of propensity score plots depicted the same ASD participant with a high propensity score (matched treatment units; this participant has a propensity score of approximately 0.8) without a close match among the selected matches in the TD group (matched control units). We next examined matching of propensity scores based on cutoff values proposed in the literature [10, 48]: a maximum standardized mean difference (*d*) of approximately .25 and variance ratios (vr) within the range of .5 to 2. The *d* value was close to the maximum of .25, although the vr was within the acceptable range (*d* = .24, vr = 1.46). Given the high standardized mean difference and the outlier seen in the propensity score plot, we removed the outlier ASD participant and conducted nearest neighbor and optimal matching methods with a revised sample of 24 children with ASD and 43 TD children.

The second iteration using the revised sample resulted in 24 children with ASD matched to 24 TD children. In this iteration, there was a difference between the two methods in the set of TD children selected for the matched group, which was a difference in one child. Propensity score distributions did not indicate clear outliers with either method. However, an examination of standardized mean differences and variance ratios indicated that the optimal matching method was better than the nearest neighbor results (optimal: *d* = .14, vr = 1.05; nearest neighbor: *d* = .24, vr = 1.32). These findings revealed that this difference in one child meant that the optimal matching method with 24 children per group resulted in better balanced groups versus the nearest neighbor method, as well as the matched groups in the first iteration with 25 ASD and 25 TD children.

The final step in diagnosing groups is to determine how well groups are matched on each covariate included in the propensity score, as well as any other pre-existing variables that may be of interest in the study [9, 32, 57]. Prior to determining propensity scores, the only variable where we expected group differences was on language variables, which we decided a priori not to incorporate in matching as discussed above. Guidelines to evaluate well-matched groups on each variable included examination of boxplots (where one would observe significant overlap when groups are well-balanced), *p* values > .5, Cohen’s *d* close to 0, and variance ratios close to 1 [3, 4, 11]. Cohen’s *d* was calculated using the compute.es package [58] with formulas in line with Kover and Atwood [4]. The use of Cohen’s *d* and variance ratios is recommended as alternatives to inferential statistics such as *p* values, due to difficulties with establishing equivalence with inferential statistics [4].

**Fig. 4:**
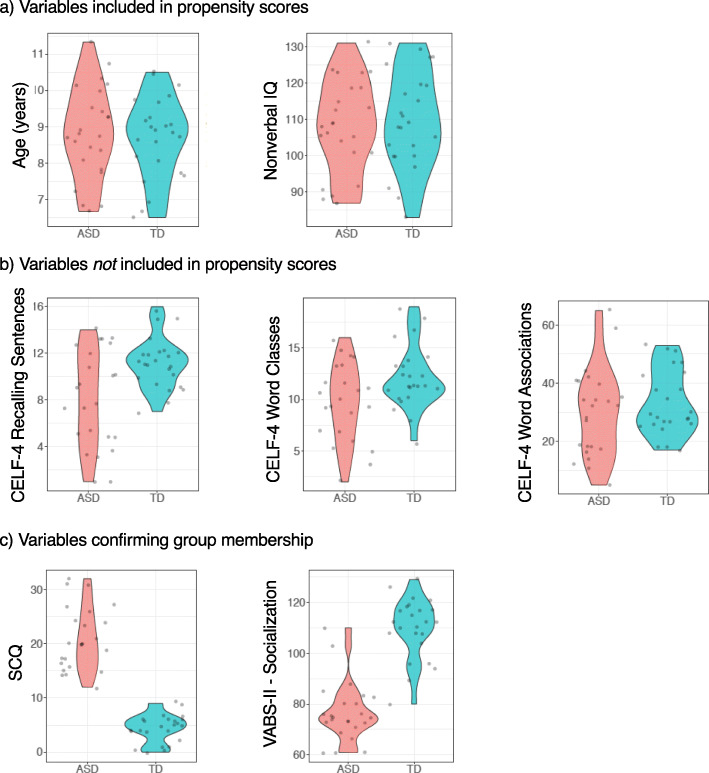
Violin plots for continuous demographic variables in final matched groups. Points represent observations per participant. For age and nonverbal IQ, matching was achieved according to criteria of *p* > .5, Cohen’s *d* close to 0, and variance ratios close to 1. CELF-4 Word Associations did not meet the criteria of *p* > .5, but distributions on this variable appear similar between groups. Groups are significantly different on other language measures of CELF-4 Recalling Sentences and Word Classes, as well as the Social Communication Questionnaire (SCQ) and the Vineland (VABS-II) Socialization Domain

As seen in Table 3 and Fig. 4, our final revised sample with the optimal matching method resulted in two successfully balanced groups according to criteria listed above on our covariates of interest, age and IQ. We next examined other variables not included in our propensity scores [32], but may be related to group diagnosis and/or performance on outcome measures. The matched groups also met the cutoff for *p* values > .5 on the ratio of English- to French-speaking children. On measures of sex, parental education level, and CELF-4 Word Association, groups were not significantly different (*p*’s between .136 and .461), but these values did not meet recommended matching cutoffs of *p* > .5. As expected prior to selecting participants, groups were significantly different in their distribution on language measures of Recalling Sentences and Word Classes. Additionally, as expected due to diagnoses, groups were significantly different on social skills measures of the SCQ and VABS-II Socialization domain.

**Table 3:** Descriptive statistics for final matched groups (ASD *n* = 24; TD *n* = 24)

	ASD (*n* = 24)	TD (*n* = 24)	*p*	*d*	vr
English- and French-dominant speaking children (En to Fr)	11:13	10:14	1		
Block order (order 1 to order 2)	12:12	13:11	1		
Age^a^	8.83 (1.26)	8.70 (1.12)	.713	.11	1.27
Nonverbal IQ^a^	108.29 (12.65)	109.50 (13.24)	.748	− .09	.91
CELF-4 Word Associations^a^	29.92 (15.01)	33.29 (11.17)	.382	− .26	1.80
Sex (M to F)	21:3	18:6	.461		
Parental education (below to above university)^c^	12:12	6:18	.136		
CELF-4, Word Classes Total^a, b^	9.74 (3.74)	12.08 (3.06)	.024*	− .69	1.49
CELF-4, Recalling Sentences^a^	8.08 (4.16)	11.17 (2.18)	.003**	− .93	3.64
Vineland Socialization subscale^a^	76.83 (11.64)	110.00 (11.88)	< .001***	− 2.82	0.96
Social Communication Questionnaire^a^	20.88 (5.83)	4.42 (2.62)	< .001***	3.64	4.95

Variables are sorted in descending order based on *p* values

Continuous and categorical variables were analyzed using paired sample *t* tests and Fisher’s exact tests, respectively

Negative values for Cohen’s *d* indicate higher values in the TD group

*d* Cohen’s *d*, *vr* variance ratio

*p < .05, **p < .01, ***p < .001

^a^The values shown are the mean (SD)

^b^One child with ASD did not complete this measure

^c^For all children, this is based on the mother except for one TD child where the mother’s education was not provided; thus, the father’s education was used instead

Lastly, we verified the distribution of children for randomized experimental factors (i.e., block order) [59]. As seen in Table 3 above including descriptive statistics, the same proportion of children had both block orders. In sum, the optimal method with 24 ASD and 24 TD established balanced groups on the desired covariates of age and IQ, and our examination of other variables helps us better understand the relative distributions of pre-existing characteristics in both groups.

### Exploratory: matching on age, IQ, and language

**Fig. 5:**
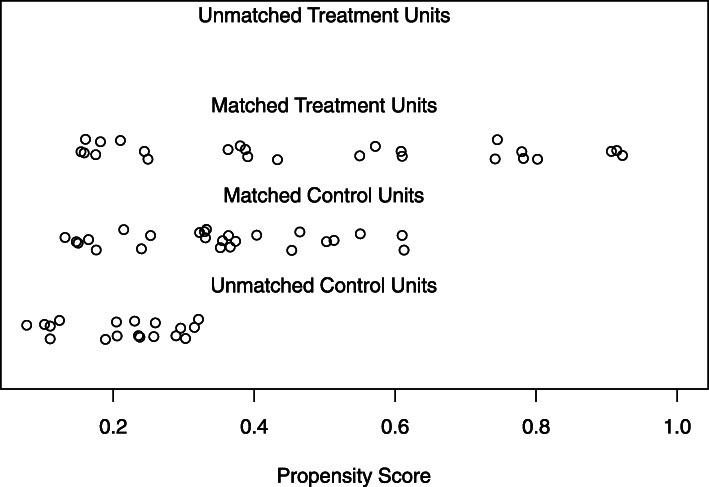
Distribution of propensity scores when including age, nonverbal IQ, and CELF-4 Recalling Sentences with the full sample (ASD = 25, TD = 43). Matched treatment units = children with ASD; matched control units = selected matches of TD children; unmatched control units = remaining unmatched TD children. This plot indicates the distribution of propensity scores when including covariates of age, IQ, and CELF-4 Recalling Sentences for all 25 children with ASD and 43 TD children. Propensity scores were calculated using the nearest neighbor method. There are 8 children with ASD who appear to be outliers relative to the propensity scores for TD children

To examine the consequences of matching on age, nonverbal IQ, and language, we conducted the nearest neighbor and optimal matching methods with all three variables. We chose the Recalling Sentences subtest to represent our language variable, because on Word Classes, one child with ASD was unable to complete the measure, and on Word Associations, the distribution between groups in the full sample appeared similar (see Fig. 2). Including all three variables of age, nonverbal IQ, and language resulted in different sets of TD children as potential matches for the 25 children with ASD with either the nearest neighbor or the optimal matching method. When using three covariates, the selected groups with both matching methods resulted in less well-balanced matches than when using just two covariates of age and IQ. For example, as seen in Fig. 5, an examination of the distribution of propensity scores with the optimal matching method demonstrated that 8 children with ASD were outside the range of propensity scores relative to the rest of the children with ASD and TD children. Table 4 describes the descriptive and inferential statistics of propensity scores and each covariate when including age, nonverbal IQ, and CELF-4 Recalling Sentences. Only the variable of age meets the desired cutoff of *p* > .5, and the distribution of CELF-4 Recalling Sentences still appears substantially different between both groups (propensity score Cohen’s *d* > .5, propensity score variance ratios > 3, and *p*s < .15 on two of three variables).

**Table 4:** Comparison between ASD and TD groups on age, nonverbal IQ, and CELF-4 Recalling Sentences (ASD = 25, TD = 25)

		Nearest neighbor		Optimal	
		Cohen’s *d*	vr	Cohen’s *d*	vr
Propensity scores		.55	3.57	.55	3.72
	ASD (*n* = 25)	TD (*n* = 25)	*p*	TD (*n* = 25)	*p*
Age (years)	8.93 (1.34)	8.73 (1.24)	.587	8.69 (1.31)	.520
Nonverbal IQ (Leiter)	107.16 (13.61)	113.00 (14.89)	.154	113.80 (15.10)	.109
CELF-4 Recalling Sentences	7.84 (4.25)	10.00 (2.10)	.029	9.88 (1.94)	.036

Due to the poor balancing when including all three proposed covariates, this evidence supports balancing on two covariates of age and nonverbal IQ to retain as many children in the sample as possible. Additionally, because language abilities of children with ASD were not categorically poorer across all three measures relative to TD children, it is unclear on which language measure to match when intercorrelations between language measures ranged widely across the full sample (rs = .28–.72), within ASD (rs = .34–.84), and within TD children (rs = .09–.47). Therefore, matching groups on two covariates of age and nonverbal IQ appear to be both theoretically supported based on prior studies and empirically supported by the current evidence with our sample.

## Discussion

This article presents a tutorial on the use of a transparent workflow that can be used to help think through and systematically document how participants are selected to create matched groups. In the spirit of the open science movement [60–62], this article provides a reproducible and replicable workflow to move towards clearer documentation of the early step of participant selection/matching prior to data analysis. A template of this workflow and accompanying questions to consider is freely available in the Additional file 1. We also provide open access to our sample dataset and accompanying R code that demonstrates the use of propensity scores as one method to conduct and document matching.

### Incorporating propensity scores into your research

There is a growing interest in the use of propensity scores in research with individuals with NDDs [12, 28, 63]. While there are multiple uses for propensity scores [10, 29, 31], we demonstrated how they can facilitate group matching when considering matching on two or more variables. Part of the relative success of using propensity scores with this sample was because we recruited participants in consideration of our goal to match groups on multiple variables known to be related to referential gaze following, word learning, and action understanding. Given known heterogeneity on IQ [64], the proposed age range, and sex ratio in ASD, we constrained recruitment to children with ASD without intellectual disability (screened first through a phone interview asking parents to characterize if the child’s verbal ability was severely delayed, delayed, or age appropriate), more boys than girls among typically developing children, and children in both groups who were 6 to 11 years of age, in addition to inclusion and exclusion criteria noted above. These restrictions limit generalization of findings to the broader population, but when implemented with justified control groups and balancing criteria, provide a first step to understanding at the very least one subgroup of individuals within a clinical population. Matching is not always a perfect method, but it is one possible method to address questions concerning individuals with NDDs.

Though this article uses propensity score analysis in R, the application of propensity scores is not limited to R. There are multiple options across other commonly used software [42], including software that does not require time to learn a coding language (e.g., SPSS). If researchers are still unsure of using propensity scores to select matched groups, written detail for each step of this same workflow can be provided in prose on free repositories such as the Open Science Framework (www.osf.io). While methods sections in published articles are meant to provide information allowing replication by future studies, often important detail must be omitted for word count and for content relevant to primary research questions. With options such as free repositories or supplemental materials/appendices depending on the journal, there are multiple options for researchers to provide detailed documentation that can further elaborate on methods. One benefit of free repositories is that they include digital object identifiers that can be cited for such work.

### Limitations

One of the major challenges in research on NDDs is that samples (along with small sample sizes) may not reflect the true heterogeneity of the population in question. This is an important consideration and is also related to larger discussions in the research community regarding recruitment and inclusion [65]. While two covariates common to NDD research were chosen for this particular sample, the method of “controlling for” covariates obscure deeper issues in NDD research such as whether those are in fact the primary covariates that should be considered, if there are omitted or unknown covariates, and how to grapple with the wide heterogeneity seen across multiple characteristics, among others. One way to include rather than constrain heterogeneity is to move towards a dimensional framework, encompassing “normal to abnormal” variation to better elucidate the nature of this heterogeneity across a full spectrum of individuals [66]. This framework may be better suited for some research questions versus others, and whether matched group designs are best suited for the research questions at hand deserves consideration during study planning. Further discussions of the challenges with matching have been discussed at length in prior work and are still relevant to current research on NDDs [11, 67, 68].

In addition to matching, there are many other methods that can be used in combination with or in contrast to matching to better understand the performance of individuals with neurodevelopmental disorders. In regression-based methods [69], one can standardize the performance of one group of individuals with NDDs against that of a comparison group. In contrast to matching, a regression-based method retains all participants, thus does not run the risk of excluding subjects that do not result in balanced matched groups. Depending on the research questions, study design, and sampling considerations (e.g., wide versus a narrow age range), one can consider from multiple options to assess the performance of individuals with NDDs relative to comparison groups. Additionally, some questions pertaining to individuals with NDDs may not even require a comparison group, and instead can focus on within-group variability. At best, it is important to decide on a method prior to data collection, but if necessary, to re-evaluate other possibilities as data collection progresses.

## Conclusion

The selection of matched groups is an important methodological consideration for quasi-experimental designs that already face challenges of data interpretation given the lack of random assignment. In research with individuals with NDDs, additional challenges of data interpretation include how to generalize results from samples that do not fully represent the heterogeneity seen in the broader population. Therefore, it is critical to ensure reproducible and replicable steps in the creation of matched groups. We demonstrate our proposed workflow to encourage clear documentation of this process and show how researchers can use propensity scores as one way to provide a transparent and reproducible matching method when selecting participants. These efforts can advance our ability to replicate research findings that can in turn inform researchers, clinicians, and educators that work with individuals with NDDs

## Supplementary information

**Additional file 1:.** Appendix. Template of the workflow.

## Data Availability

The datasets analyzed during the current study are available in a public repository, https://github.com/janetybang/propensity_scores. A tutorial of the current workflow is available at https://janetybang.github.io/propensity_scores/. The template of the workflow is available in the attached Additional file 1 as well as at https://osf.io/6a3e4/

## Footnotes

1 This simplified example ignores many assumptions regarding sampling, design, and blinding, which are among many of the important factors for determining high-quality randomized control trials.

2 *n* = number of items, *C* = combinations, *r* = number of items being chosen at a time where *n*!/*r*!(*n* − *r*)!. For example, if we wanted to get all possible sets of 2 from a sample of 4 then the formula is: 4!/2! (4 − 2)! = 4!/2! × 2! = (4 × 3 × 2 × 1)/(2 × 1) × (2 × 1) = 24/4 = 6

3 There is a theoretical basis for matching on propensity scores being akin to random assignment [29], but we would strongly caution against this interpretation given that the combination of wide heterogeneity and small sample sizes in research with NDDs may limit our ability to account for numerous unknown covariates. Users should fully inform themselves of this background and whether the appropriate assumptions were met in order to interpret causality [32, 33].

4 Two groups may also share the same NDD, but differ in their distribution on one variable, for example, comparing children with autism spectrum disorder who have low language abilities versus those with higher language abilities [45], or a randomized control trial comparing outcomes of children with ASD who are undergoing treatment (intervention group) versus those who are not undergoing the same treatment (control group).

5 We thank a reviewer for this helpful example.
