# Supplementary material for: Towards greater transparency in neurodevelopmental disorders research: use of a proposed workflow and propensity scores to facilitate selection of matched groups
Source: J Neurodev Disord. 2020 Jul 24;12:20. doi: 10.1186/s11689-020-09321-6 (PMC7382075; doi:10.1186/s11689-020-09321-6)
Supplement: Supplementary file 1 — Additional file 1:. Appendix. Template of the workflow. [file 11689_2020_9321_MOESM1_ESM.docx]

Appendix

**Workflow to Achieve Matched Groups - TEMPLATE**

Bang, Sharda, & Nadig

Last revised: May 31, 2020

The steps below were created to assist with establishing matched groups. We welcome researchers to use these steps as a template or develop their own depending on their own study questions and design.

We encourage researchers to make any version of this process a shared resource for all team members to access and edit. It would be ideal to implement this workflow as a working draft during study planning, especially prior to data analyses of study outcome variables. However, it can still be a useful resource at the stage of exploratory analyses or re-analysis of data.

Importantly, we also encourage researchers to share their processes on free repositories (e.g., the Open Science Framework; www.osf.io), such that others can use it as a guide or reference for their own work. If you would like to share your workflow(s) here, please contact us and we would be happy to use this repository to include a variety of examples.

A similar workflow using propensity scores is available at https://janetybang.github.io/propensity_scores/.

Study name: ________________________________________________

Authors: ___________________________________________________

1. **ASSESS** data

The final possible sample size to include is X children in group 1 and X children in group 2.

Document the decisions that inform the final statement above such as:

- How many participants do not meet study criteria? What are the reasons for exclusion?
- How many children were tested in pilot testing (e.g., how do we draw the line for which children are considered as a part of pilot testing or not?)
- Are there any children excluded due to other study requirements (e.g., minimum number of observations, passing practice trials)?

1. **SELECT** covariates

We will include the following covariates to match groups:

1. Covariate 1. The rationale to match on covariate 1 is _______ (CITE).
2. Covariate 2. The rationale to match on covariate 2 is _______ (CITE).

Document the decisions that inform the final statement above such as:

- Will we restrict heterogeneity during recruitment to accommodate primary matching covariates (e.g., age) and/or secondary variables we intend to be similar between groups (e.g., females vs. males)?
- What other covariates were also considered? Why were they excluded from matching?
- If applicable, what covariates will be explored in post-hoc covariate analyses?

1. **CONDUCT** matching

We chose to conduct matching using _________ (e.g., the _______ method in the

MatchIt package in R, hand selection).

Document the decisions that inform the final statement above such as:

- Was the person who hand-selected the matches blinded to information of participants?
- What steps were taken to organize the values to facilitate matching (e.g., first, values on covariate 1 were sorted in descending order for each group).
- What distance unit was decided upon to pairwise matching (e.g., individual in group 2 was included in the matched group if it was within X units of a value for an individual in group 1).
- How were matches selected when there were two of the same value for either group?
- What covariates were considered first if two or more are included in matching?

1. **DIAGNOSE** data

Our final matched sample includes ____ individuals in group 1 and _____ individuals in group 2. This was achieved on iteration X.

Our critieria for achieving matched groups are detailed below. Descriptive and inferential statistics were confirmed through visual inspection of __________ (e.g., one or more of the following: histograms, boxplots).

- *p* > .50
- variance ratios close to 1
- Cohen’s d close to 0
- Others?

Iteration 1: Results and Decisions

Iteration 2: Results and Decisions

Iteration 3: Results and Decisions

Document the decisions that inform the final statement above such as:

- What are the results of each iteration?
- If you do decide to iterate, what is the rationale to iterate each time (e.g., *p* values were close to 0 and Cohen’s *d* was more than 1 SD).
